# *POLD2* and *KSP37* (*FGFBP2*) Correlate Strongly with Histology, Stage and Outcome in Ovarian Carcinomas

**DOI:** 10.1371/journal.pone.0013837

**Published:** 2010-11-04

**Authors:** Bente Vilming Elgaaen, Kari Bente Foss Haug, Junbai Wang, Ole Kristoffer Olstad, Dario Fortunati, Mathias Onsrud, Anne Cathrine Staff, Torill Sauer, Kaare M. Gautvik

**Affiliations:** 1 Department of Gynaecology, Oslo University Hospital, Oslo, Norway; 2 Department of Medical Biochemistry, Oslo University Hospital, Oslo, Norway; 3 Department of Pathology, Oslo University Hospital, Oslo, Norway; 4 Department of Gynaecology, Oslo University Hospital, Oslo, Norway; 5 Department of Pathology, Oslo University Hospital, Oslo, Norway; 6 Department of Medical Biochemistry, Oslo University Hospital, Oslo, Norway; 7 Faculty of Medicine, University of Oslo, Oslo, Norway; Bauer Research Foundation, United States of America

## Abstract

**Background:**

Epithelial ovarian cancer (EOC) constitutes more than 90% of ovarian cancers and is associated with high mortality. EOC comprises a heterogeneous group of tumours, and the causes and molecular pathology are essentially unknown. Improved insight into the molecular characteristics of the different subgroups of EOC is urgently needed, and should eventually lead to earlier diagnosis as well as more individualized and effective treatments. Previously, we reported a limited number of mRNAs strongly upregulated in human osteosarcomas and other malignancies, and six were selected to be tested for a possible association with three subgroups of ovarian carcinomas and clinical parameters.

**Methodology/Principal Findings:**

The six selected mRNAs were quantified by RT-qPCR in biopsies from eleven poorly differentiated serous carcinomas (PDSC, stage III–IV), twelve moderately differentiated serous carcinomas (MDSC, stage III–IV) and eight clear cell carcinomas (CCC, stage I–IV) of the ovary. Superficial scrapings from six normal ovaries (SNO), as well as biopsies from three normal ovaries (BNO) and three benign ovarian cysts (BBOC) were analyzed for comparison. The gene expression level was related to the histological and clinical parameters of human ovarian carcinoma samples. One of the mRNAs, DNA polymerase delta 2 small subunit (*POLD2*), was increased in average 2.5- to almost 20-fold in MDSC and PDSC, respectively, paralleling the degree of dedifferentiation and concordant with a poor prognosis. Except for *POLD2*, the serous carcinomas showed a similar transcription profile, being clearly different from CCC. Another mRNA, Killer-specific secretory protein of 37 kDa (*KSP37*) showed six- to eight-fold higher levels in CCC stage I compared with the more advanced staged carcinomas, and correlated positively with an improved clinical outcome.

**Conclusions/Significance:**

We have identified two biomarkers which are markedly upregulated in two subgroups of ovarian carcinomas and are also associated with stage and outcome. The results suggest that *POLD2* and *KSP37* might be potential prognostic biomarkers.

## Introduction

In Norway and the United States, ovarian cancer is the fourth and fifth most frequent cause of cancer death in women, respectively [1], [2]. At the time of diagnosis, almost 70% of the patients have distant spread of disease (stage III–IV), and their 5-year relative survival rate is only about 30% [1], [2]. The cause(s) and mode of progression are poorly understood, and the patients are treated similarly in spite of tumour heterogeneity [3]–[6].

EOC comprises several subtypes of histopathologically different tumours [7]. There is growing evidence for the existence of at least two distinct tumourigenetic pathways, corresponding to the development of type I and type II tumours [3], [6], [8]–[10]. Type I tumours include highly differentiated serous carcinomas, mucinous carcinomas, endometroid carcinomas, clear cell carcinomas and malignant Brenner tumours. They are thought to arise from precursor lesions such as cystadenomas, borderline tumours or endometriosis and suggested to be a result of mutations in e.g. KRAS, BRAF, CTNNB1 or PTEN genes [4], [6], [8], [9]. Type II carcinomas include moderately and poorly differentiated serous carcinomas, carcinosarcomas and undifferentiated carcinomas, and appear to originate *de novo* from as yet no known identified precursor lesions, possibly resulting from mutations in e.g. TP53 [4], [6], [8], [9], [11]. Thus, ovarian carcinogenesis appears to be associated with abnormalities in multiple gene families. How these genetic alterations are reflected in changes in transcriptional activity and carcinogenesis are not understood.

Previously, we reported a limited number of mRNAs strongly upregulated in human osteosarcomas and several other malignancies [12]. Further analyses on various types of human malignant cell lines and normal tissues showed that six mRNAs were highly expressed: *KSP37*, *C9orf89*, *PRAT4A*, *NOLA2*, *ANT2* and *POLD2* (Table 1). Apart from *C9orf89* and *PRAT4A* (unknown at project start), these mRNAs code for proteins known to be associated with malignancy [13]–[16]. We hypothesized that these mRNAs might as well be associated with ovarian cancer. In the present study, we quantified these mRNAs by RT-qPCR in biopsies from eleven PDSC (stage III–IV), twelve MDSC (stage III–IV) and eight CCC (stage I–IV) as well as control tissue representing six SNO, three BNO and three BBOC. The expression levels were related to histological, clinical and laboratory parameters. We found that two of the mRNAs were markedly upregulated in two subgroups of ovarian carcinomas and also associated with stage and outcome.

**Table 1:** Title and assumed function of six selected mRNAs [12].

Title	Assumed function
Killer-specific secretory protein of 37 kDa; *KSP37*	Cytotoxic lymphocyte-mediated immunity [13]
Chromosome 9 open reading frame 89; *C9orf89*	CARD binding region* [29]
Protein associated with TLR4,A; *PRAT4A*	TLR4 associated* [30]
Nucleolar protein family A, member 2; *NOLA2*	Associated with telomerase and snoRNPs [14]
Adenine nucleotide translocator 2; *ANT2*	ADP/ATP exchange [15]
DNA polymerase delta 2 small subunit; *POLD2*	DNA replication and repair [16]

* Unknown at project start. CARD: Caspase Recruitment Domain. TLR: Toll-like receptor. SnoRNPs: small nucleolar ribonucleoproteins. *KSP37* is synonymous with fibroblast growth factor binding protein 2; *FGFBP2* (www.ncbi.nlm.nih.gov/genebank).

## Results

### Mean expression levels of six selected mRNAs in three subgroups of ovarian carcinomas compared with three different control groups

**Figure 1:**
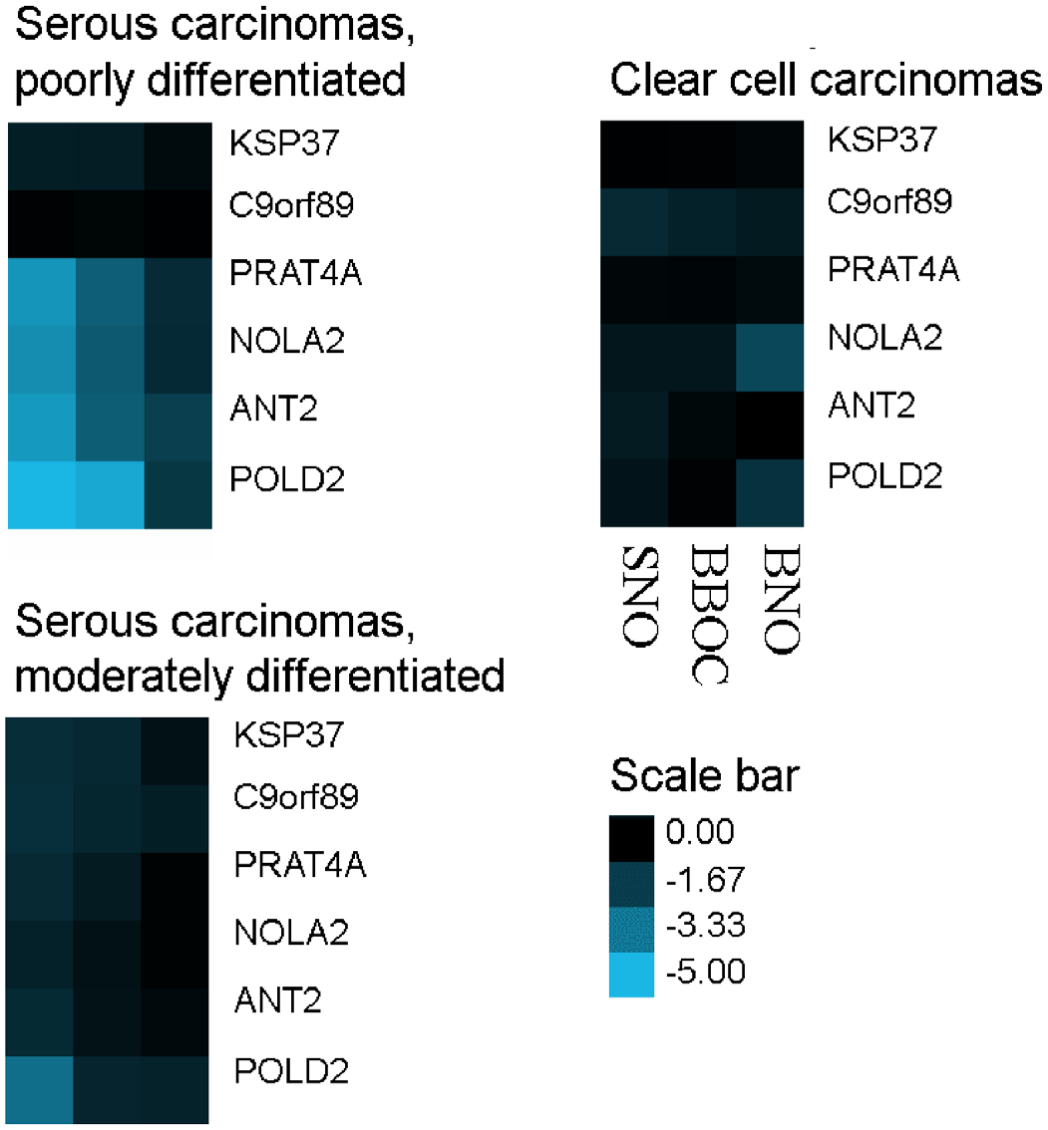
Mean differential expression levels of six selected mRNAs (horizontal) in three subgroups of ovarian carcinomas compared with three different control tissues (vertical). Log10 p-values of the T-test of delta Cq values are shown as heat-maps, where the smaller the p-value, the brighter the blue colour (scale bar). P<0.05 represents significant differential expression. SNO: superficial scrapings from normal ovaries. BBOC: biopsies from benign ovarian cysts. BNO: biopsies from normal ovaries.

**Figure 2:**
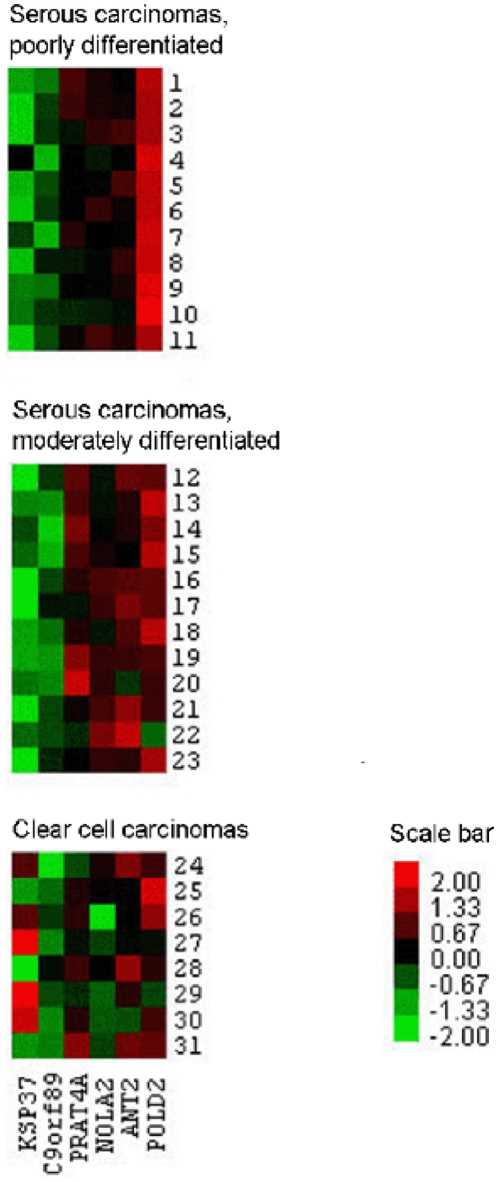
Differential expression levels of six selected mRNAs (vertical) in 31 individual tissue samples (horizontal) of three subgroups of ovarian carcinomas compared with superficial scrapings from normal ovaries. Normalized log_2_ transformed original FC values (Z-scores) are shown as heat-maps, where the higher/lower the FC value, the brighter the red/green colour, respectively (scale bar). Black colour illustrates no difference in FC values of cancer tissue and control tissue.

**Figure 3:**
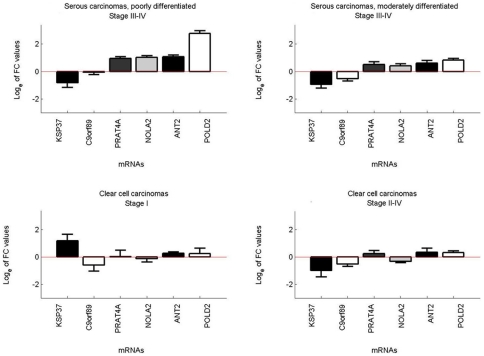
Mean expression levels of six selected mRNAs in moderately and poorly differentiated serous carcinomas (stage III–IV) and clear cell carcinomas (stage I and II–IV) compared with superficial scrapings from normal ovaries. Log_e_ transformed original FC values with standard deviation are shown as bar plots.

Expression levels of the six selected mRNAs in PDSC, MDSC and CCC are presented in Figures 1,2,3. Figure 1 shows heat-maps of log10 transformed p-values (t-test) comparing the mean expression levels as ΔCq (delta quantification cycles) values in PDSC, MDSC and CCC with SNO, BNO and BBOC. P-values less than 0.05 were used as cut-off value for significance. When comparing PDSC with SNO and BBOC, respectively, the following mRNAs were significantly differentially expressed: *PRAT4A* (p = 8.1×10^−5^ and 2.6×10^−3^), *NOLA2* (p = 1.3×10^−4^ and 3.5×10^−3^), *ANT2* (p = 6.3×10^−5^ and 2.6×10^−3^) and *POLD2* (p = 3.4×10^−8^ and 2.4×10^−5^), whereas comparing these carcinomas with BNO, *ANT2* (p = 1.9×10^−2^) and *POLD2* (p = 3.1×10^−2^) showed a differential expression. For MDSC, *POLD2* (p = 9.1×10^−4^) showed differential transcription when compared with SNO. *NOLA2* (p = 1.1×10^−2^) and *POLD2* (p = 4.3×10^−2^) were differentially expressed when CCC were compared with BNO. These significantly differentially expressed mRNAs were all upregulated in PDSC and MDSC, while downregulated in CCC (data not shown). Thus, several of the six previously shown upregulated mRNAs in osteosarcomas were also differentially expressed in the ovarian carcinomas. Furthermore, the overall transcriptional activity of these genes was similar when comparing BBOC with SNO and BNO, while *PRAT4A* and *POLD2* showed significant differential expression (p<0.05) when BNO and SNO were compared (data not shown).

### Individual expression levels of six selected mRNAs in three subgroups of ovarian carcinomas compared with SNO controls

Figure 2 shows mRNA expression profiles of all 31 carcinomas employing SNO as a control group, depicted as heat-maps of normalized log_2_ transformed original fold change (FC) values. Higher mRNA levels were detected in PDSC and MDSC for *PRAT4A*, *NOLA2*, *ANT2* and *POLD2*. *PRAT4A*, *NOLA2* and *ANT2* showed a similar mRNA expression in PDSC and MDSC in contrast to *POLD2*, being clearly more upregulated in PDSC compared with MDSC. The mRNA levels were reduced for *KSP37* and *C9orf89* in both PDSC and MDSC. Furthermore, except for *C9orf89*, a distinct mRNA expression pattern of the mRNAs was present in CCC. The heat-maps looked almost identical when BBOC were used as the control group, but differed slightly when BNO were used (Figure S1).

### Mean expression levels of six selected mRNAs in three subgroups of ovarian carcinomas compared with SNO controls

Figure 3 shows bar plots of mean mRNA expression (log_e_ transformed original FC values) in PDSC, MDSC and different stages of CCC, using SNO for comparison. In PDSC, *POLD2* was almost 20-fold upregulated (FC 19.4), whereas *C9orf89*, *PRAT4A*, *NOLA2* and *ANT2* were only moderately upregulated (FC 1.2–3.1) and *KSP37* slightly downregulated (FC 0.7). In MDSC, transcription levels of *KSP37* and *C9orf89* were reduced (FC 0.5 and 0.7 respectively), while the other mRNAs showed moderate upregulations (FC 1.8–2.5). In CCC stage I, *KSP37* was markedly upregulated (FC 4.3), but downregulated in the more advanced stages of CCC (FC 0.5). In both stage I and stages II–IV of CCC, *PRAT4A*, *ANT2* and *POLD2* were slightly upregulated (FC 1.4–1.7), whereas *C9orf89* and *NOLA2* were slightly downregulated (FC 0.6–0.96). Thus, when comparing *KSP37* expression levels in CCC stage I with the more advanced stages of CCC, MDSC and PDSC, a six- to eight-fold difference was detected. Further analyses of the FC values in Figure 3 are shown in Table 2. The mean mRNA profiles were almost identical when BBOC were used as control tissue, but differed more when BNO were used (Figure S2).

**Table 2:** Statistical analyses of the FC values shown in Figure 3.

	KSP37	C9orf89	PRAT4A	NOLA2	ANT2	POLD2
PDSC, stage III–IV						
Average	0.70	1.17	2.77	3.03	3.12	19.42
Stdev	0.62	0.93	1.06	1.43	1.11	14.79
Min	0.07	0.50	1.24	1.36	1.51	5.90
Max	1.64	3.27	4.82	6.73	4.92	59.30
MDSC, stage III–IV						
Average	0.52	0.69	1.98	1.78	2.40	2.50
Stdev	0.39	0.46	1.15	1.42	2.36	1.13
Min	0.06	0.29	0.60	0.74	0.77	1.38
Max	1.42	1.79	3.97	6.06	9.42	4.66
CCC, stage I						
Average	4.28	0.69	1.42	0.96	1.35	1.66
Stdev	3.11	0.46	1.32	0.50	0.28	1.54
Min	0.95	0.17	0.39	0.57	1.17	0.67
Max	8.40	1.29	3.34	1.68	1.77	3.94
CCC, stage II–IV						
Average	0.49	0.64	1.38	0.73	1.60	1.43
Stdev	0.40	0.23	0.65	0.12	0.91	0.35
Min	0.12	0.36	0.90	0.56	0.82	1.11
Max	1.04	0.90	2.31	0.83	2.76	1.75

PDSC: Poorly differentiated serous carcinomas. MDSC: Moderately differentiated serous carcinomas. CCC: Clear cell carcinomas. Stdev: Standard deviation. Min: minimal value. Max: Maximal value. mRNA description is given in Table 1.

The mean mRNA expressions, given as log_e_ transformed original FC values, in the different ovarian carcinoma subgroups were also compared (t-test). P-values less than 0.001 were used as cut-off value for significance. *POLD2* mRNA levels were significantly higher in PDSC compared with both MDSC (FC 19.4 vs. 2.5; p = 1.7×10^−8^) and CCC (FC 19.4 vs. 1.5; p = 5.6×10^−8^), whereas transcription levels of *NOLA2* and *ANT2* were significantly higher in PDSC compared with CCC (FC 3.0 vs. 0.8; p = 3.0×10^−6^ and FC 3.1 vs. 1.5; p = 5.7×10^−4^, respectively). The results were similar irrespectively of the control tissue used (data not shown).

### Correlation of mRNA expression to clinical, laboratory and histological parameters

In a single-factor linear regression model, normalized FC values of the six mRNAs, employing SNO as controls, were correlated with clinical, laboratory and histological parameters. The parameters shown in Table S1 as well as histological subgroups were included in the regression analysis. The significant positive correlations (p<0.05) between mRNA expression levels and these parameters are shown in Table 3. Only *KSP37* was significantly associated with several clinical parameters, being positively associated with favourable prognostic factors such as localized disease, long progression-free survival (>18 months) and long overall survival (>36 months). Furthermore, it was negatively associated with unfavourable prognostic factors such as more advanced disease, short progression-free survival and short overall survival (data not shown). When correlating the FC values with histological subgroups, *KSP37* expression was positively associated with CCC, whereas *PRAT4A*, *NOLA2* and *POLD2* were positively associated with PDSC. The transcriptional levels of *C9orf89* and *ANT2* did not correlate with any of the parameters.

**Table 3:** Results of single-factor regression analysis.

	KSP37	PRAT4A	NOLA2	POLD2
Clinical parameters				
FIGO stage I (all CCC)	7.9×10^−7^			
Progression-free survival ≥18 months	1.6×10^−2^			
Overall survival ≥36 months	3.3×10^−2^			
Status at last follow-up: Alive, no relapse of EOC	8.0×10^−5^			
Status at last follow-up: Alive, relapse of EOC		1.2×10^−2^		
Histological parameters				
PDSC		1.8×10^−2^	2.1×10^−3^	1.2×10^−5^
CCC	6.8×10^−3^			

CCC: Clear cell carcinomas. PDSC: Poorly differentiated serous carcinomas. EOC: Epithelial ovarian cancer. Significant positive correlations (p-values) between mRNA expression levels and parameters are shown. Detailed explanation is given in Table 1 and Table S1.

## Discussion

A major finding in this study was the strong upregulation of *POLD2* in PDSC compared to control tissues and other histological subgroups of ovarian carcinomas examined. *POLD2* is a subunit of the DNA polymerase delta complex, encoding a protein involved in DNA replication and repair [16]. It is downregulated by the PTEN tumour suppressor gene [17], already known to be involved in ovarian carcinogenesis [4], [6], [8], [9]. In gliomas, a consistent pattern of chromosomal alterations were found involving altered regions which harboured seven “landscape genes” associated with patient survival, among these *POLD2* [18].

*KSP37* mRNA levels were clearly and distinctly regulated in early stage of CCC, another histological subgroup of ovarian cancer. *KSP37* is identified as *FGFBP2*, a member of the fibroblast growth factor binding protein 2 family (www.ncbi.nlm.nih.gov/genebank). It is expressed in cytotoxic T lymphocytes and natural killer cells, and is suggested to have a “cytotoxic potential” which so far has not been identified [13]. Yamanaka et al. found that a high *KSP37* expression in high-grade gliomas was positively correlated with survival. Furthermore, *KSP37* was more closely correlated with survival than histological grade [19], while in the present study, a positive correlation with histological type, clinical stage as well as good prognosis was observed.

A challenge related to the understanding of molecular portraits of ovarian cancer has been the lack of representative control tissue. Histologically, EOC is thought to originate from the single layer of ovarian surface epithelium (OSE) [5], [7], [20]–[22], which therefore should be the most representative control tissue. Because the OSE represents only a small fraction of the total ovary, the availability of OSE RNA is limited. Zorn et al [23] compared the gene expression profiles of OSE brushings, whole ovary samples, cultures of normal OSE and immortalized OSE cell lines. The transcriptional profiles were markedly distinct, but it was concluded that OSE brushings were most representative as control material, since it is not exposed to *in vitro* manipulations and does not contain stromal components. In the present study, OSE, as represented by six superficial scrapings from normal ovaries (SNO) was used as reference material. Furthermore, three biopsies from normal ovaries (BNO) and three biopsies from benign ovarian cysts (BBOC) were included for additional comparisons. Our results showed that the investigated six mRNAs were similarly expressed in SNO and BBOC, but differed more in BNO (data not shown). Furthermore, the mRNA levels of the carcinomas were similar both when compared to SNO and BBOC, but different when compared to BNO (Figures 1,2,3 and Figure S1, S2). Apparently, SNO and BBOC showed comparable transcriptional activity for these six mRNAs. The findings are not unexpected, since the benign ovarian cysts used for control tissue are believed to originate from OSE, whereas BNO mainly consist of stromal tissue [7]. Thus, for study purposes, benign cysts originating from OSE, being simpler to obtain than OSE, and superficial scrapings of normal ovaries appear to be alternative choices as control tissue for EOC.

Except for the marked upregulation of *POLD2* in PDSC, the expression levels of the other mRNAs in PDSC and MDSC were similar, in agreement with a common tumourigenetic pathway for moderately and poorly differentiated serous carcinomas as previously suggested [10]. Thus, the fact that *POLD2* mRNA expression paralleled the dedifferentiation of MDSC to PDSC, increasing from 2.5-fold in MDSC to almost 20-fold in PDSC, underscores the uniqueness of this transcript. Since patients with PDSC generally have a worse clinical outcome than patients with MDSC, the significantly higher *POLD2* expression in PDSC compared with MDSC could have a bearing on a poor prognosis, possibly through a replication advantage in cells overexpressing POLD2.

The marked upregulation of *KSP37* confined to CCC stage I, as well as its positive association with clinical variables of good prognosis, suggest also a possible predictive role of this transcript. Even though these results are very much in concordance with overall results from studies on other malignancies, the present results are novel related to ovarian carcinomas and need to be confirmed. The different transcriptional profiles for clear cell carcinomas and serous carcinomas are in agreement with distinct tumourigenetic pathways for these carcinomas and also consistent with other studies [24], [25]. Although the present study is based on a limited patient cohort of only three subgroups of ovarian carcinomas, the strong association of two of the mRNAs with histology, stage and outcome suggest that they may have potential as cancer markers.

## Materials and Methods

### Patients and tissue material

The study was approved by the Regional Committee of Medical and Health Research Ethics (REK) in Eastern Norway and all participating women signed informed consent. Tissue specimens as well as clinical and laboratory information were obtained from women primarily operated for gynecological tumours at Oslo University Hospital, Ulleval, in the period 2003 to 2008. All tissue samples were snap-frozen in liquid nitrogen, except SNO, which were transferred to 500 µl TRIzol solution (Invitrogen.com) immediately after harvesting in order to avoid mRNA degradation. The samples were stored in a biobank at −80°C until processed.

The expression of the six selected mRNAs was studied in a total of 31 epithelial ovarian carcinomas and twelve benign samples. The carcinomas included twelve MDSC (stage III–IV), eleven PDSC (stage III–IV) and eight CCC (stage I–IV). Six SNO, three BNO and three BBOC were used for comparison. SNO were taken from the surface of normal ovaries by scraping the ovaries with a scalpel, as cervical pap smear brushings yielded too little material (data not shown). By this method, the vast majority of harvested cells were immunologically verified as epithelial (data not shown). The three benign cysts were cystadenofibromas, containing both epithelial and stromal cells. BNO consisted almost exclusively of stromal cells as confirmed by histology. In accordance with the literature [23], we used OSE, represented by SNO, as reference material. The histological diagnoses of all samples were confirmed by an experienced pathologist.

Clinical and laboratory information was collected from hospital records and additional preoperative patient interviews, shown in Table S1. All patients and controls were of Western European descent, postmenopausal (apart from two being perimenopausal) and had no diseases influencing survival other than the ovarian cancer. All patients but four (two with MDSC and two with PDSC) were primarily operated by at least a total hysterectomy or a uterus amputation, a bilateral salpingo-oophorectomy and an omentectomy. No patients received neoadjuvant chemotherapy, whereas all patients but three (one in each histological group) received adjuvant chemotherapy. The effect of treatment was evaluated by clinical examinations and serum CA125 measurements at minimum.

### Selected mRNAs

Six mRNAs were selected from a subtraction cDNA library of human osteosarcoma [12]. They represented interesting candidate genes, being strongly upregulated in several osteosarcoma and other malignant human cell lines, and showed a differential expression between human cancers and normal tissues. Except for C9orf89 and PRAT4A, whose identities and functions were unknown at project start, these mRNAs code for proteins possibly associated with malignancies. The titles and assumed protein functions of the selected candidate mRNAs are shown in Table 1.

### Primer sequences

PCR primers (Table S2) were designed by using the Invitrogen database and tested for homology with other sequences at the NCBI gene website (www.ncbi.nlm.nih.gov). All primers were intron spanning to avoid co-amplification of genomic DNA.

### RNA isolation

Tissue specimens were either crushed frozen or homogenized directly for 2×2 minutes in 750 µl TRIzol using a Tissuelyzer (Qiagen.com). Total RNA was extracted using the TRIzol method according to the manufacturer's instructions. Isolated total RNA was quantified (Nano Drop spectrophotometer, Saveen Werner AB) and quality controlled by the RNA Nano 6000 assay on the Bioanalyzer 2100 system (Agilent). RNA integrity number (RIN) and 28S/18S ratios were calculated to ensure a satisfactory RNA quality and integrity of the samples. To remove genomic DNA, total RNA was treated using RNase-free DNase I (Roche.com). Total RNA was further purified on RNeasy MinElute clean up spin columns (Qiagen.com), eluted with RNase free water, aliquoted and stored at −80°C until analyzed.

### Quantitative reverse transcription-polymerase chain reaction (RT-qPCR)

One µg of total RNA from each sample was reversely transcribed using 2.5 U/µl Omniscript enzyme (Qiagen.com), 1 X RT-buffer, 1 mM dNTPs, 2.5 µM oligo-d(T)-primer and 1 U/µl RNase inhibitor (final concentrations) in a total volume of 20 µl for one hour at 37°C. For all samples, a negative RT-control without Reverse Transcriptase enzyme was included. cDNA was PCR-amplified with primers from the six specific mRNAs and two endogenous reference genes (β-actin and GAPDH) in replicate sets of two to six, with a coefficient of variation of less than 1.6 percent. The samples were analyzed on a real-time fluorescence LightCycler instrument (Roche.com) according to the manufacturer's instructions in a final volume of 20 µl using a LightCycler Fast start SYBR Green kit. PCR conditions essentially contained 2 µl cDNA, 25 mM MgCl_2_ and 0.5 µM of forward and reverse primers. The following cycle conditions were used: 10 min denaturation at 95°C before 45 cycles at 95°C for 0 s, 56°C for 10 s and 70°C for 5 s.

Gene expression patterns for the six selected mRNAs were calculated using the comparative crossing threshold method of relative quantification (ΔΔCq method) [26], and presented as relative (ΔCq) and fold change (FC) values. All expression levels were normalized to the reference genes separately, giving overall similar results. β-actin quantification was most linear over a wide dilution range and preferred as reference gene. ΔCq was designated as the mean quantification cycle of an mRNA in a tissue subtracted with the mean quantification cycle of a reference RNA in the same tissue. ΔΔCq was calculated as mean ΔCq of each of the three different control groups subtracted by ΔCq of each cancer tissue sample (mean of replicates), whereas FC was 2^ΔΔCq^.

### Statistical analysis

Mean ΔCq values of each histological subgroup of ovarian carcinomas were compared to mean ΔCq values of each control group by performing a two-tailed t-test, presented in heat-maps by log10 transformed p-values (Figure 1). Log_2_ transformed original FC values of each individual sample (n = 31) were normalized (Z-scores) and shown as heat-maps by applying a two-way clustering method [27] (Figure 2 and Figure S1). Mean original FC values of the three ovarian carcinoma subgroups were presented by log_e_ transformed bar plots (Figure 3 and Figure S2). Finally, a linear regression model [28], testing the correlation of histological, clinical and laboratory parameters with mRNA expression levels given as normalized FC values, was used (Table 3).

## Supporting Information

Figure S1 Differential expression levels of six selected mRNAs (vertical) in 31 individual tissue samples (horizontal) of three subgroups of ovarian carcinomas compared with biopsies from benign ovarian cysts (a) and biopsies from normal ovaries (b). Normalized log2 transformed original FC values (Z-scores) are shown as heat-maps, where the higher/lower the FC value, the brighter the red/green color, respectively (scale bar). Black color illustrates no difference in FC values of cancer tissue and control tissue.
(1.92 MB TIF)

Figure S2 Mean expression levels of six selected mRNAs in moderately and poorly differentiated serous carcinomas (stage III–IV) and clear cell carcinomas (stage I and II–IV) compared with biopsies from benign ovarian cysts (a) and biopsies from normal ovaries (b). Loge transformed original FC values with standard deviation are shown as bar plots.
(2.74 MB TIF)

Table S1 Clinical and laboratory information for patients included.
(0.05 MB DOC)

Table S2 Primer sequences of six selected mRNAs.
(0.04 MB DOC)
