# Correction: *POLD2* and *KSP37* (*FGFBP2*) Correlate Strongly with Histology, Stage and Outcome in Ovarian Carcinomas

**DOI:** 10.1371/annotation/a9e87423-6f0f-4a0b-be01-7125f0a41109

**Published:** 2010-11-11

**Authors:** Bente Vilming Elgaaen, Kari Bente Foss Haug, Junbai Wang, Ole Kristoffer Olstad, Dario Fortunati, Mathias Onsrud, Anne Cathrine Staff, Torill Sauer, Kaare M. Gautvik

The correct affiliations are:

Bente Vilming Elgaaen 1,2*, Kari Bente Foss Haug 3#, Junbai Wang 4#, Ole Kristoffer Olstad 3, Dario Fortunati 3, Mathias Onsrud 2,5, Anne Cathrine Staff 2,5, Torill Sauer 4,5, Kaare M. Gautvik 3,5

1 Department of Gynaecological Oncology, Oslo University Hospital, Oslo

Norway**

2 Department of Gynaecology, Oslo University Hospital, Oslo, Norway

3 Department of Medical Biochemistry, Oslo University Hospital, Oslo, Norway

4 Department of Pathology, Oslo University Hospital, Oslo, Norway

5 Faculty of Medicine, University of Oslo, Oslo, Norway

*corresponding author

# These authors contributed equally to this work.

**Current address.

