# Supplementary figures and images for: POLD2 and KSP37 (FGFBP2) Correlate Strongly with Histology, Stage and Outcome in Ovarian Carcinomas
Source: PLoS One. 2010 Nov 4;5(11):e13837. doi: 10.1371/journal.pone.0013837 (PMC2973954; doi:10.1371/journal.pone.0013837)

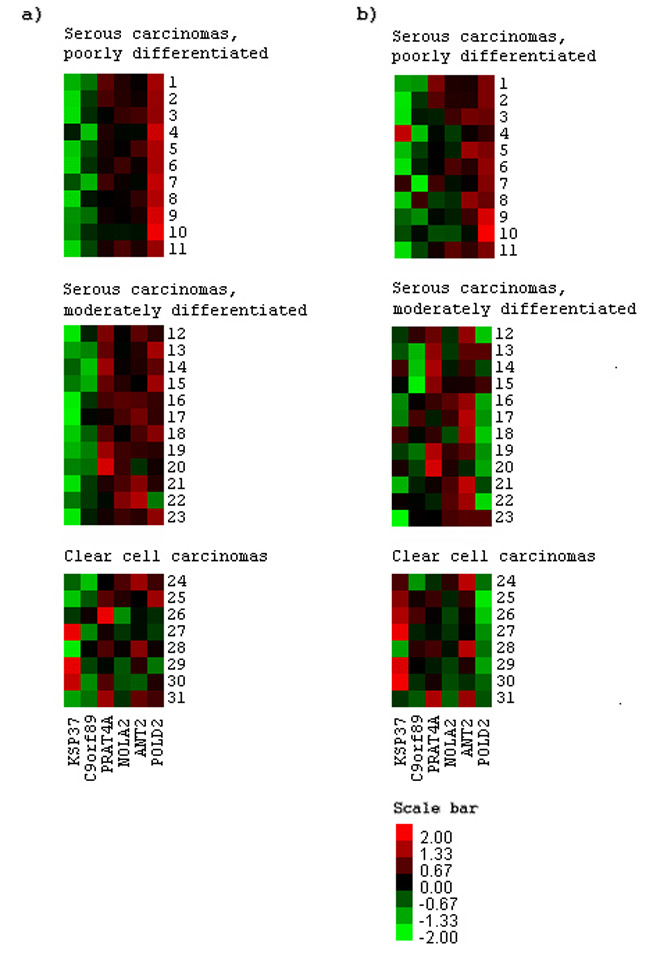

Supplement: Figure S1 — Differential expression levels of six selected mRNAs (vertical) in 31 individual tissue samples (horizontal) of three subgroups of ovarian carcinomas compared with biopsies from benign ovarian cysts (a) and biopsies from normal ovaries (b). Normalized log2 transformed original FC values (Z-scores) are shown as heat-maps, where the higher/lower the FC value, the brighter the red/green color, respectively (scale bar). Black color illustrates no difference in FC values of cancer tissue and control tissue. (1.92 MB TIF) [file pone.0013837.s001.tif]

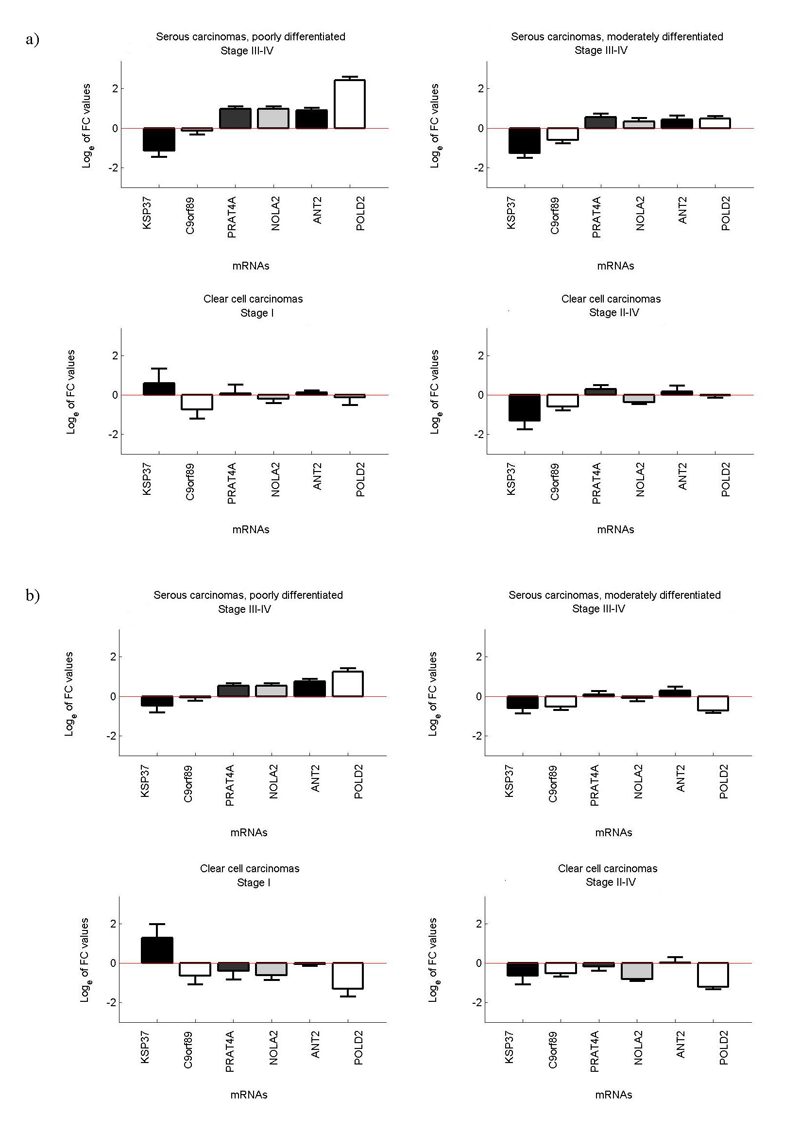

Supplement: Figure S2 — Mean expression levels of six selected mRNAs in moderately and poorly differentiated serous carcinomas (stage III–IV) and clear cell carcinomas (stage I and II–IV) compared with biopsies from benign ovarian cysts (a) and biopsies from normal ovaries (b). Loge transformed original FC values with standard deviation are shown as bar plots. (2.74 MB TIF) [file pone.0013837.s002.tif]
