# Supplementary material for: POLD2 and KSP37 (FGFBP2) Correlate Strongly with Histology, Stage and Outcome in Ovarian Carcinomas
Source: PLoS One. 2010 Nov 4;5(11):e13837. doi: 10.1371/journal.pone.0013837 (PMC2973954; doi:10.1371/journal.pone.0013837)
Supplement: Table S1 — Clinical and laboratory information for patients included. (0.05 MB DOC) [file pone.0013837.s003.doc]

Table S1. Clinical and laboratory information for patients included.

| Parameters | MDSC (n=12) | PDSC (n=11) | CCC (n=8) |
| --- | --- | --- | --- |
| Age ≥ 65 | n=6 | n=5 | n=4 |
| Preoperative condition |  |  |  |
| Good | n=11 | n=11 | n=7 |
| Poor^a^ | n=1 | n=0 | n=1 |
| Preoperative CA 125 |  |  |  |
| <35 kU/L (normal) |  |  | n=1 |
| 35-500 kU/L | n=3 | n=4 | n=3 |
| >500 kU/L | n=9 | n=7 | n=4 |
| FIGO stage |  |  |  |
| I |  |  | n=4 (1 IA, 3 IC) |
| II |  |  | n=1 (IIA) |
| III | n=10 (IIIC) | n=8 (1IIIA, 7IIIC) | n=2 (IIIC) |
| IV | n=2 | n=3 | n=1 |
| Start of chemotherapy^b^ |  |  |  |
| < 28 days after surgery | n=3 | n=3 | n=1 |
| ≥ 28 days after surgery | n=8 | n=7 | n=6 |
| Standard chemotherapy treatment^c^ | n=5 | n=9 | n=4 |
| Optimal CA 125 normalization^d^ | n=5 (of 11) | n=8 (of 10) | n=5 (of 6)^e^ |
| Progression-free survival^f^ |  |  |  |
| <18 months | n=10 | n=7 | n=3 |
| ≥18 months | n=2 | n=4 | n=5 |
| Overall survival |  |  |  |
| <36 months | n=9 | n=6 | n=4 |
| ≥36 months | n=3 | n=5 | n=4 |
| Status at last follow-up |  |  |  |
| Alive, no EOC | n=1 | n=1 | n=4 |
| Alive, with EOC |  | n=2 |  |
| Dead of EOC | n=11 | n=7 | n=4 |
| Dead of other disease |  | n=1 |  |

MDSC: Moderately differentiated serous carcinomas. PDSC: Poorly differentiated serous carcinomas. CCC: Clear cell carcinomas. EOC: Epithelial ovarian cancer. ^a^: ascites ≥50 ml at surgery, haemoglobin <10 g/dL and albumin<36 g/L. ^b^: one patient in each group did not receive chemotherapy. ^c^: four to nine cycles of Carboplatine and Paclitaxel. ^d^: normalization of CA125 (< 35 kU/L) within four cycles of chemotherapy. ^e^: two patients had normal preoperative CA 125. ^f^: progression: Doubling of pathologic CA 125 levels or clinical relapse.
