# Supplementary material for: POLD2 and KSP37 (FGFBP2) Correlate Strongly with Histology, Stage and Outcome in Ovarian Carcinomas
Source: PLoS One. 2010 Nov 4;5(11):e13837. doi: 10.1371/journal.pone.0013837 (PMC2973954; doi:10.1371/journal.pone.0013837)
Supplement: Table S2 — Primer sequences of six selected mRNAs. (0.04 MB DOC) [file pone.0013837.s004.doc]

Table S2. Primer sequences of six selected mRNAs.

| mRNAs | Primer sequences |
| --- | --- |
| *KSP37*, fw | 5´- TGG GAA CAT TGT TGG AAA CC -3´ |
| *KSP37*, rv | 5´- GGT TGT CTG TCA GGG AGA GG -3´ |
| *C9orf89*, fw | 5´- GTA CTG CTA TCC GCC AGA CC -3´ |
| *C9orf89*, rv | 5´- CAG GAA GGC CAG CAG GTA G -3´ |
| *PRAT4A*, fw | 5´- AGA GGT GGC TGA CCT CAA GA -3´ |
| *PRAT4A*, rv | 5´- AGG TCT TCC TCC TGG TGG TT -3´ |
| *NOLA2*, fw | 5´- TTT TGG CAG GAG ACA CAC TG -3´ |
| *NOLA2*, rv | 5´- CAC CCA GGT CCG TCT TAG AG -3´ |
| *ANT2*, fw | 5´- ATC TAC CGA GCC GCC TAC TT -3´ |
| *ANT2*, rv | 5´- ATC CAG CTG ATG ACG ATG TG -3´ |
| *POLD2*, fw | 5´- TCC AAA TGA GAC CCT TCC TG -3´ |
| *POLD2*, rv | 5´- CCA CAC AGC ACT TCT CCT CA -3´ |
